# Immobilization of Lead Migrating from Contaminated Soil in Rhizosphere Soil of Barley (*Hordeum vulgare* L.) and Hairy Vetch (*Vicia villosa*) Using Hydroxyapatite

**DOI:** 10.3390/ijerph14101273

**Published:** 2017-10-23

**Authors:** Masahiko Katoh, Elsya Risky, Takeshi Sato

**Affiliations:** 1 Department of Agricultural Chemistry, School of Agriculture, Meiji University, Kawasaki, Kanagawa 214-8571, Japan; 2 Department of Civil Engineering, Graduate School of Engineering, Gifu University, Gifu 501-1193, Japan; elsyarisky@yahoo.com; 3 Department of Civil Engineering, Faculty of Engineering, Gifu University, Gifu 501-1193, Japan; tsat@gifu-u.ac.jp

**Keywords:** hydroxyapatite, lead immobilization, mobility, rhizosphere, tolerance to lead toxicity

## Abstract

This study conducted plant growth tests using a rhizobox system to quantitatively determine the distance of immobilization lead migrating from contaminated soil into uncontaminated rhizosphere soil, and to assess the lead phases accumulated in rhizosphere soil by sequential extraction. Without the hydroxyapatite, exchangeable lead fractions increased as the rhizosphere soil got closer to the contaminated soil. Exchangeable lead fractions were higher even in the rhizosphere soil that shares a boundary with the root surface than in the soil before being planted. Thus, plant growth of hairy vetch was lower in the soil without the hydroxyapatite than in the soil with the hydroxyapatite. The presence of hydroxyapatite may immobilize the majority of lead migrating from contaminated soil into the rhizosphere soil within 1 mm from the contaminated soil. The dominant lead fraction in the rhizosphere soil with the hydroxyapatite was residual. Thus, plant growth was not suppressed and the lead concentration of the plant shoot remained at the background level. These results indicate that the presence of hydroxyapatite in the rhizosphere soil at 5% wt may immobilize most of the lead migrating into the rhizosphere soil within 1 mm from the contaminated soil, resulting in the prevention of lead migration toward the root surface.

## 1. Introduction

Lead poses risks to humans and animals, as well as to plant growth; thus, lead contamination in soil and water environments is a common and harmful worldwide concern. In particular, heavy lead soil contamination exists near mines [1] and shooting ranges [2]. Soil remediation techniques should be applied to such sites to protect environmental and public health from lead migration through the soil profile into groundwater and its uptake by root systems. In these areas, lead contamination is extensive and these areas have very low asset value. Therefore, typical treatments, including soil washing and excavation and transportation to landfill sites, are not suitable because of high management and conveyance costs.

Phytostabilization, which stabilizes toxic metals in the root zone and prevents their leaching into groundwater [3,4], is one of the phytoremedial approaches [5]. Phytostabilization is an eco-friendly and cost-effective technique; thus, it seems to be a suitable approach for heavily and extensively lead-contaminated soil. However, phytostabilization has certain limitations. For example, some toxic metals are absorbed by the plant, and the presence of the plant may induce the enhancement of the concentration of metals in the soil solution [3]. However, phytostabilization combined with immobilization can compensate for these limitations and can greatly reduce the mobility of toxic metals, such as cadmium, lead, and zinc [6,7,8], although the application of iron grit, which is used as an amendment to lower metal leaching, could enhance cadmium and lead concentrations in plants [9]. The bioavailability of lead can be reduced by immobilization materials; thus, the plant growth is enhanced, which further enhances the stabilization of toxic metals [3,6,10].

Hydroxyapatite (Ca_5_(PO_4_)_3_OH) is expected to be utilized as an immobilization material for phytostabilization combined with lead immobilization. This is because hydroxyapatite can effectively immobilize lead even in soils with very high concentrations [11]. Hydroxyapatite primarily immobilizes lead by precipitating pyromorphite [12]. The lead of pyromorphite has been reported to hardly re-dissolve [13,14,15,16,17] when compared with the lead immobilized by other mechanisms of hydroxyapatite such as ion exchange and surface complexation [18,19]. However, to enhance the reliability of phytostabilization combined with the hydroxyapatite, the reaction and stability of lead in the soil, particularly those of the rhizosphere soil, should be deeply understood. This is because even when phytostabilization combined with immobilization, the extractability, and leachability of toxic metals including lead may become high in the planted soil [4,20]. Some studies have investigated lead immobilization in bulk soil [21,22], as well as metal availability and the chemical properties of rhizosphere soil without immobilization materials [23,24]. In lead-contaminated rhizosphere soil applied with hydroxyapatite, Katoh et al. [20] investigated the stability of lead immobilized, and observed that the amount of water-extractable lead in the buckwheat rhizosphere soil 1 mm apart from the root surface increased to the same level as that in the non-planted soil without the hydroxyapatite. However, to the best of our knowledge, it remains unclear how lead migrates from contaminated soil and accumulates into the rhizosphere soil with hydroxyapatite during cultivation as compared with rhizosphere soil without hydroxyapatite.

To this end, we conducted plant growth experiments using a rhizobox system in which the rhizosphere soil can be accurately collected in amounts as small as 1 mm [20]. The aim of this study was to quantitatively determine the distance of immobilization and the accumulation of lead migrating from the contaminated soil, and the phases of lead accumulated as compared with rhizosphere soil without hydroxyapatite. This study provides valuable information for understanding how lead migrates from contaminated soil and immobilizes in the rhizosphere soil, and how plant growth is prevented from lead toxicity by phytostabilization combined with hydroxyapatite. These would be useful when applying phytostabilization combined with hydroxyapatite.

## 2. Materials and Methods

### 2.1. Preparation of Soil and Apatite

The lead-contaminated soils used in the present study were collected at depths of 5–15 cm from a shooting range in Tajimi located in Gifu, Japan (137°06′5″–137°29′2″E). The area of the shooting range had a mean annual precipitation of 2200 mm and a mean temperature of 14 °C. The soil sample was air-dried, and passed through a 2-mm sieve prior to chemical analysis and cultivation tests. The total lead content in the soil was 25.7 mg kg^−1^, and the pH was 7.2 (Table 1). The contaminated soil had a light clay texture. Commercial soil was used as the non-contaminated soil (Table 1), and its soil texture was similar to the contaminated soil. Hydroxyapatite was used as the immobilization material because it can effectively immobilize lead by precipitating lead phosphate minerals, such as pyromorphite [25]. The hydroxyapatite was synthesized from gypsum and diammonium hydrogen phosphate [26], passed through a 0.425-mm sieve, and used as the immobilization material. Hydroxyapatite synthesized by this procedure can effectively immobilize lead [11], and its capacity of lead sorption has been reported to be approximately 562 mg g^−1^ [27].

### 2.2. Cultivation Using a Rhizobox System

The plant cultivation tests were conducted by using a 130(L) × 165(W) × 80(H) mm rhizobox system [20,23]. The details of the rhizobox system have been described in Katoh et al. [20]. In brief, the system comprised three compartments: The plant compartment (PC), rhizosphere compartments (RCs), and the side compartments (SCs). The PC comprised of a 3-mm-thick U-shaped frame, which had the same length and height as the rhizobox. The PC was separated from other compartments using a 25 μm nylon mesh, and it was placed at the center of the box. The RCs comprised of three hollow shaped frames. Each frame was 1-mm-thick and was the same length and height as the box, and the RCs were separated from each frame and other compartments by using the nylon mesh. The RCs were placed on both sides of the PC; the compartment nearest to the PC was RC3, the second was RC2, and the farthest was RC1. The SCs were the space not occupied by the PC and RCs in the box. We added 46.1 g and 15.4 g × 3 of non-contaminated soil to the PC and each RCs, respectively, and 605 g of contaminated soil to each SC. The non-contaminated soil of RCs was well mixed with and without hydroxyapatite at 5% wt, and was packed into the RC. This amount of material was calculated to be sufficient to immobilize total lead in the contaminated soil, and was based on a preliminary sorption experiment [27]. Ultra-pure water was added in order to maintain 50% of the soil’s water-holding capacity during the test period. Three seedlings were transplanted to the PC one week after germination using the non-contaminated soil. The side and upper parts of the rhizobox were covered with aluminum foil to provide shade except for the upper part of the PC. The water content in the soil was maintained at its initial level by the addition of water every two or three days. The evapotranspiration rate of the plants was calculated as the sum of the amount of water added. The plants were grown for two months at 25 °C (room temperature) with a 14 h photoperiod using a 40 W horticultural lamp. Barley (*Hordeum vulgare* L.) and hairy vetch (*Vicia villosa*) were selected and grown in the PC of the rhizobox system for the plant cultivation test, because both of the plants have a very weak tolerance to lead toxicity [28,29]. Three boxes were prepared for each treatment.

After two months of plant cultivation, the above-ground plants were cut and the shoots were dried and weighed. The total lead concentration in the dried shoots was determined to calculate the lead uptake in the shoots. The soil samples in the RCs were carefully collected from the boxes, and no roots were found in the soil sample in the RCs. The soil samples in the RCs were defined as rhizosphere soil [20]. This is because the surface of the PC could be considered as the surface of the plant root [20]. The collected soil samples were air-dried and passed through a 1 or 0.425 mm sieve before chemical analysis.

### 2.3. Analytical Methods

Soil texture was determined using the hydrometer method [30]. Soil pH was measured in ultra-pure water using a pH meter (MM-60M, DKK-TOA Co., Tokyo, Japan). The total carbon content of the soil was determined using a carbon, hydrogen, and nitrogen (CHN) elemental analyzer (MT-6; Yanaco New Science Inc., Tokyo, Japan). The amount of water-extractable lead and organic carbon were determined by ultra-pure water extraction at a liquid/solid (LS) ratio of 10 using inductively coupled plasma atomic emission spectrometry (ICP-AES; ULTIMA2; Horiba Ltd., Kyoto, Japan), and a total organic carbon analyzer (TOC-V_WS_; Shimadzu Co., Kyoto, Japan). The total lead and phosphorus contents in the soil and plant samples were determined by acid digestion with HNO_3_ and HCl using a microwave digestion system. The amorphous iron was extracted following the method of Shuman [31]. A sequential extraction procedure was performed on the soil samples following the method by Tessier et al. [32]. In brief, each fraction was extracted with a 1 M MgCl_2_ solution (exchangeable fraction), 1 M sodium acetate solution at pH 5 (carbonate fraction), and 0.04 M NH_2_OH-HCl in 25% (*v*/*v*) acetic acid solution in a water bath at 95 °C with occasional agitation (Fe/Mn oxide fraction), 0.02 M HNO_3_ solution and 5 mL 30% H_2_O_2_ solution in a water bath at 85 °C with occasional agitation (organic fraction), and 5 mL of HNO_3_ and 2 mL of HCl using a microwave digestion system (residual fraction). All of the extracted or digested solutions were passed through a 0.45 μm filter and were analyzed to determine the lead concentration using ICP-AES.

### 2.4. Statistical Analyses

Statistical analysis was performed using JMP Ver. 8.0.2 (SAS Institute Inc., Cary, NC, USA). The difference between pairs of means for the evapotranspiration, weight, lead concentration, and lead uptake of the plant shoots with and without the hydroxyapatite was identified using Student’s *t*-test. Analysis of variance (ANOVA) was performed to compare soil pH, water-extractable lead in the soil, and total summed content of the lead fraction in the sequential extraction samples of the rhizosphere soil. The differences between mean values were determined using Tukey’s honestly significant difference (HSD) test at a 95% confidence level.

## 3. Results

### 3.1. Shoot Biomass and Lead Uptake from Contaminated Soil

The shoot weights of barley and hairy vetch grown in the soil with the RC with the hydroxyapatite were higher than those without the hydroxyapatite. However, the shoot weight of barley was not noted to be significantly different (Table 2). The lead concentrations of plant shoots were the opposite; those were lower in the plants with the RC with the hydroxyapatite at 7 and 9 μg g^−1^ for barley and hairy vetch, respectively, than without the hydroxyapatite. However, there were no significant differences in lead concentrations between with and without the hydroxyapatite groups for both plants due to the high coefficients of variation of the lead concentrations in the plants without the hydroxyapatite. The evapotranspiration rates of the plants were 40 to 66 mm for barley and 65 to 70 mm for hairy vetch.

### 3.2. Soil pH and Water-Extractable Lead in Rhizosphere Soil

**Figure 1:**
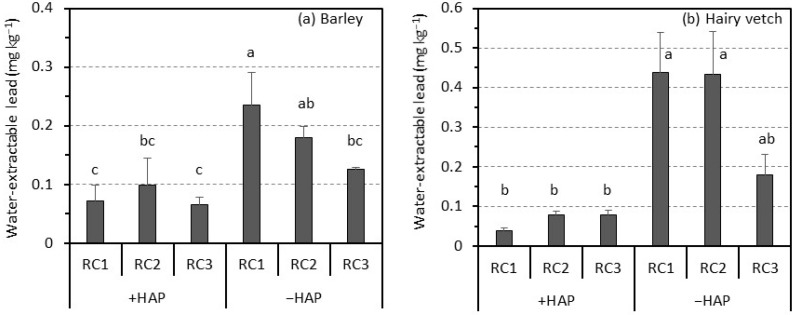
Water-extractable lead in rhizosphere soil (RC1–RC3) with and without hydroxyapatite (+HAP and −HAP, respectively) for (**a**) barley and (**b**) hairy vetch. Vertical bars indicate the standard error (S.E.) (*n* = 3). Different letters indicate significant differences at *p* < 0.05 based on Tukey’s HSD test.

The soil pH in RC1 without the hydroxyapatite was 5.2 despite the plant type (Table 3), and it was compatible with that in the soil before it was planted (Table 1). Getting closer to the PC, the soil pH decreased, and the soil pH in the RC3 was 5.0 for barley and 4.9 for hairy vetch. This reduction in rhizosphere soil pH may be attributed to the root secretion, such as organic acids and their decomposition in the rhizosphere soil [20,33]. Conversely, in the same RC, the soil pH with the hydroxyapatite was higher than that without the hydroxyapatite due to the pH buffering of hydroxyapatite [34,35]. Similar to the rhizosphere soil without the hydroxyapatite, the soil pH in RC3 was significantly lower than that in the RC2 for barley and RC1 for hairy vetch. The amounts of water-extractable lead in the rhizosphere soil of RCs without the hydroxyapatite for both plants increased to more than 0.10 mg kg^−1^ (Figure 1), whereas those before planting were less than 0.02 mg kg^−1^ (Table 1). In addition, the water-extractable lead in the rhizosphere soil of RCs without the hydroxyapatite increased as the rhizosphere soil of RC got closer to the contaminated soil. The amount of water-extractable lead in the rhizosphere soil of RC1 was significantly higher than that in the rhizosphere soil of RC3 for barley. The levels of water-extractable lead in the soil of RCs with the hydroxyapatite were suppressed to 0.06–0.10 mg kg^−1^ for barley and 0.04–0.08 mg kg^−1^ for hairy vetch. The levels of water-extractable lead in the rhizosphere soil with the hydroxyapatite were not significantly different among RC1, RC2, and RC3. In addition, the levels were significantly lower than that in the rhizosphere soil of RC1 without the hydroxyapatite for both plants. However, the levels of water-extractable lead in the rhizosphere soil of RCs with the hydroxyapatite were higher than that in the soil before planting where the water-extractable lead was not detected at less than 0.02 mg kg^−1^ (Table 1).

### 3.3. Lead Phases by Sequential Extraction in Rhizosphere Soil

**Figure 2:**
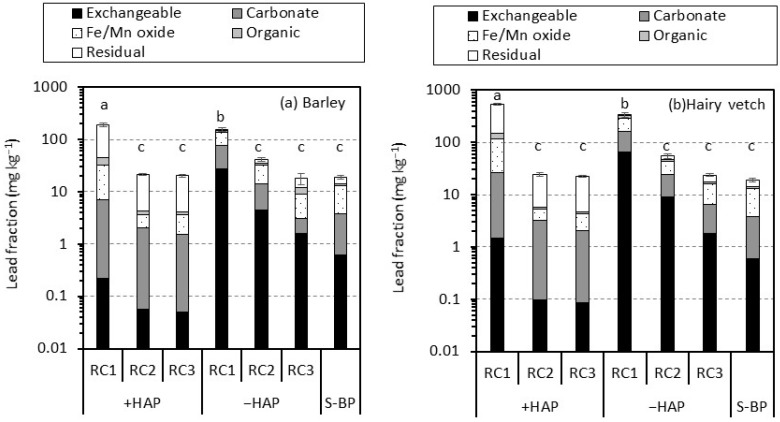
Sequential extraction of lead from soil before being planted (S-BP) and rhizosphere soil (RC1–RC3) with and without hydroxyapatite (+HAP and −HAP, respectively) for (**a**) barley and (**b**) hairy vetch. Vertical bars indicate the standard error of total lead (S.E.) (*n* = 3). Different letters indicate that the sum total content of lead fraction significantly differed at *p* < 0.05 based on Tukey’s HSD test.

Figure 2 shows the lead phases by sequential extraction in the rhizosphere soil of RCs with and without the hydroxyapatite for barley and hairy vetch. The sum total content of the lead fraction in the sequential extraction of the rhizosphere soil of RC3 for both of the plants was not significantly different from that in the soil before planting despite the addition of hydroxyapatite. That in the rhizosphere soil of RC2 with the hydroxyapatite was not significantly different from that in the soil before planting. The average value of the sum total content of the lead fraction in the rhizosphere soil of RC2 without the hydroxyapatite was higher than that in the soil before being planted, but it was not significant for both of the plants. The sum total contents of the lead fractions in the rhizosphere soil of RC1 with and without the hydroxyapatite significantly increased when compared with the soil before planting despite the plant type. Comparing the rhizosphere soil of RC1 with and without the hydroxyapatite, the presence of hydroxyapatite made the sum total content of lead significantly high regardless of plant type.

**Figure 3:**
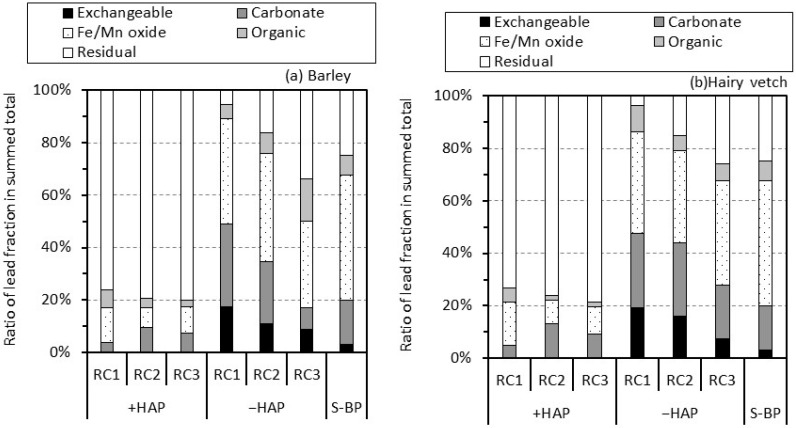
Lead fraction of the sum total by sequential extraction in soil before it was planted (S-BP) and rhizosphere soil (RC1–RC3) with and without hydroxyapatite (+HAP and −HAP, respectively) for (**a**) barley and (**b**) hairy vetch.

The composition of the soil before it was planted was as follows (Figure 3): 3.2% exchangeable fraction, 16.9% carbonate fraction, 47.5% Fe/Mn oxide fraction, 7.8% organic fraction, and 24.7% residual fraction. In the rhizosphere soil of RCs without the hydroxyapatite, the lead content and percentage in the exchangeable and carbonate fractions increased as the rhizosphere soil of RC got closer to the contaminated soil. The percentages of exchangeable and carbonate fractions in the rhizosphere soil of RC1 increased to 17.6% and 31.5% for barley and 19.3% and 28.3% for hairy vetch, respectively. In contrast, in the rhizosphere soil of RCs with the hydroxyapatite, the dominant lead fraction, was residual and ranged from 76.3% to 80.1% for barley and 73.1% to 78.7% for hairy vetch. The lead content in the exchangeable fraction in the rhizosphere soil of RC2 and RC3 with the hydroxyapatite decreased to 0.1 mg kg^−1^ for both plants as compared with the soil before it was planted (0.6 mg kg^−1^). Similarly, the lead content in the carbonate fraction of the rhizosphere soil of RC2 and RC3 with the hydroxyapatite was lower or the same level as that in the soil before it was planted. In the rhizosphere soil of RC1 with the hydroxyapatite, the sum lead content in the exchangeable and carbonate fractions increased to 7.0 mg kg^−1^ for barley and 26.7 mg kg^−1^ for hairy vetch when compared with the soil before they were planted (3.8 mg kg^−1^), while the sum lead percentage in those fractions decreased from 20.1% to 3.7% for barley and 4.9% for hairy vetch.

## 4. Discussion

### 4.1. Lead Migration from Contaminated Soil into Uncontaminated Rhizosphere Soil and Its Immobilization by Hydroxyapatite

The plant cultivation test using the rhizobox system showed that lead in the contaminated soil more than 3 mm apart from the root surface migrated into the rhizosphere soil. In particular, lead was accumulated within 1 mm from the contaminated soil, regardless of the addition of hydroxyapatite (Figure 2). However, in the RCs in which lead accumulated, the lead phases were greatly different between the rhizosphere soil with and without the hydroxyapatite (Figure 2 and Figure 3). These results suggest some insight into lead migration in rhizosphere soil. In the rhizosphere soil without the hydroxyapatite, lead would gradually migrate throughout the rhizosphere soil profile during cultivation. In addition, lead would particularly accumulate as the readily soluble phases (exchangeable and carbonate fractions) even in the rhizosphere soil (RC3) that shared a boundary with the root surface (PC). Thus, the distance that a large part of the lead migrated during cultivation would be within a few mm. These findings are consistent with results obtained by Ogawa et al. [36] who conducted the unsaturated-column-percolation test to evaluate the distance of lead migration. They reported that the distance of lead migration during 170 mm of water migration was within 5 mm. The differences in the distance noted between this study and the study by Ogawa et al. [36] would result from the amount of water migration during the test period.

In the rhizosphere soil with the hydroxyapatite, almost all of the lead that migrated from the contaminated soil accumulated in the rhizosphere soil within 1 mm from the contaminated soil. These results demonstrate that the lead that migrated from the contaminated soil was immediately immobilized by the hydroxyapatite in the rhizosphere soil. Ogawa et al. [36] estimated the range of lead immobilization by hydroxyapatite in the soil, and estimated it to be 1 mm between lead and hydroxyapatite. Thus, lead would be immobilized by the hydroxyapatite within 1-mm-thickness of rhizosphere soil. This is supported by the results of lead phases by the sequential extraction. The dominant lead fraction in the rhizosphere soil of RC1 with the hydroxyapatite was residual irrespective the plant type. More than 73% of lead was distributed into this fraction (Figure 2 and Figure 3). These results indicate that the presence of hydroxyapatite in the rhizosphere soil makes the lead phases more stable in the process of lead migration. Pyromorphite precipitated by the hydroxyapatite is a chemically stable lead compound [13,14]. The formation of pyromorphite is most likely a lead immobilization mechanism when compared with other mechanisms; thus, the pyromorphite formation would result in the alteration of lead phases to be more stable.

The levels of water-extractable lead in the rhizosphere soil of RCs with the hydroxyapatite, however, were more than 0.06 mg kg^−1^ for barley and 0.04 mg kg^−1^ for hairy vetch, while those before the soil was planted were not detected. In addition, the amount of exchangeable and carbonate lead fractions was higher in the rhizosphere soil of RC1 with the hydroxyapatite than in the soil before it was planted. These results indicate that the presence of hydroxyapatite in the rhizosphere soil at 5% wt could not completely prevent lead migration throughout the profile of rhizosphere soil, although most of the lead that migrated into the rhizosphere soil could be immobilized by the hydroxyapatite, as mentioned above. Lead would migrate in the soil pore with the water migration. The ratio of volume for hydroxyapatite to volume for the soil pore was calculated as 8.0% (*v*/*v*) on the basis of the soil and hydroxyapatite particle densities (2.7 and 3.2 g cm^−3^, respectively), the volume of RC (8.7 cm^3^), and the amounts of soil and hydroxyapatite (15.4 and 0.77 g, respectively). Thus, it is suggested that the ratio of material volume to soil pore volume in this study was low in order to completely prevent lead migration; a small amount of lead migrated throughout the profile of rhizosphere soil without the contact and reaction with the hydroxyapatite. For lead immobilization by hydroxyapatite, the material needs to be added to the contaminated soil at a stoichiometric molar ratio of more than 3/5 (=0.6) for phosphorus/lead [37]. In this study, the ratio was calculated as 2.40 on the basis of the total contents of lead in the soil (25,700 mg kg^−1^: Table 1) and phosphorus in the material (185 mg kg^−1^), as well as the addition ratio of hydroxyapatite to the soil (5% wt). Furthermore, the addition ratio of this study was considered to immobilize lead in the rhizosphere soil of RCs even if all the lead in the contaminated soil migrated into the rhizosphere soil of RCs. Therefore, this study implies that the addition of hydroxyapatite on the basis of the addition ratio calculated by the stoichiometric molar ratio and the lead immobilization capacity of the material could not prevent strict lead migration. A higher dosage of hydroxyapatite than that estimated by such methods may be required for the strict prevention of lead migration in the rhizosphere soil.

### 4.2. Plant Growth in Rhizosphere Soil with and without Hydroxyapatite in Which Lead Migrated from Contaminated Soil

The plant growth in the soil with the RC without the hydroxyapatite was reduced by the lead that migrated from the contaminated soil when compared with that in the soil with the hydroxyapatite (Table 2). Tlustoš et al. [38] and Katoh et al. [20] showed that plant growth is suppressed by lead in the rhizosphere soil less than 3 mm apart from the root surface. This study, however, demonstrated that plant growth is also suppressed by the lead migration from the contaminated soil more than 3 mm apart from the root surface as compared with that in the soil with the hydroxyapatite. This would result from lead migration toward the root surface, and the subsequent lead accumulation in the readily soluble phases near the root surface. However, the magnitude of the effect of lead toxicity depended on the individual plant, and the coefficients of variation in the lead concentrations in the plants would be higher without the hydroxyapatite than with it for both plants (Table 2). The dependence on the individual plant to lead toxicity may be explained by a small amount of uneven lead accumulation as the exchangeable phase in the rhizosphere soil that shares a boundary with the root surface. The apparent adverse effect may be observed if the cultivation period was longer.

Lead concentrations in the shoots of barley and hairy vetch grown in the soil with RC with the hydroxyapatite were within similar levels as those of plants grown in non-contaminated soil [20,39,40]. These results indicate that the plant growth in the soil with the RC with the hydroxyapatite was not reduced by the lead that migrated from the contaminated soil. This would be attributed to the immediate lead immobilization when lead migrated into the rhizosphere soil. However, the water-extractable lead was detected from the rhizosphere soil of RC3. In addition, the levels of water-extractable lead in the rhizosphere soil of RC3 were not significantly different between samples with and without the hydroxyapatite (Figure 1). Nevertheless, for both plants, plant growth with the hydroxyapatite was not reduced. This is possibly explained by the lower content of exchangeable lead in the rhizosphere soil of RC3 with the hydroxyapatite than without the hydroxyapatite (Figure 2). Namely, the higher content of exchangeable lead in the rhizosphere soil of RC3 without the hydroxyapatite than in the soil before it was planted would cause the plant growth to be inhibited, while its lower content in the rhizosphere soil with the RC3 with the hydroxyapatite did not. In addition, it is suggested that the level of water-extractable lead observed in this study (ca. 0.10 mg kg^−1^) did not directly cause the adverse effects on the plant growth. The exchangeable metal is considered as bioavailable, and the bioavailability of toxic metals is a critical factor for plant absorption [41]. This study indicates that the presence of hydroxyapatite in the rhizosphere soil at 5% wt could immobilize the lead that migrated from the contaminated soil within 1-mm from the contaminated soil, resulting in the prevention of lead migration toward the root surface. Thus, plants could grow without the adverse effects from lead toxicity. However, even in the rhizosphere soil with the hydroxyapatite, a small amount of lead is migrated and accumulated as the water-extractable phase into the rhizosphere soil that shares a boundary with the root surface. This suggests that there is a possibility that migrated lead reduces plant growth even in the soil with the hydroxyapatite. Katoh et al. [20] pointed out that when applying phytostabilization combined with hydroxyapatite to lead-contaminated soil, the plants that cannot re-mobilize lead should be selected. In addition to that, our results suggest that the plants displaying a tolerance of lead toxicity should be selected for phytostabilization combined with hydroxyapatite. If plants with a low tolerance of lead susceptibility are selected, the dosage of material should increase in order to strictly prevent lead migration in the rhizosphere soil.

## 5. Conclusions

In the rhizosphere soil of RCs without the hydroxyapatite for both barley and hairy vetch, as the rhizosphere soil of RC got closer to the contaminated soil, the lead content and percentage in the exchangeable and carbonates fractions by the sequential extraction increased when compared with those in the soil before it was planted. Thus, the distance that a large part of the lead migrated during cultivation was within a few mm. Barley and hairy vetch do not have a high tolerance to lead toxicity. The plant growth of hairy vetch was lower in the soil with the RC without the hydroxyapatite than in the soil with the RC with the hydroxyapatite. The lead that migrated from the contaminated soil 3 mm apart from the root surface potentially caused the plant growth to become low, but the lead concentrations of the plant shoots were not significantly different between the presence and absence of hydroxyapatite. This would be attributed to a small amount of uneven lead accumulation at the exchangeable phase around the root surface. In the rhizosphere soil of RCs with the hydroxyapatite, most of the lead that migrated from the contaminated soil was immobilized within 1-mm rhizosphere soil from the contaminated soil. The dominant lead fraction in the soil of RCs with the hydroxyapatite was residual. Therefore, the presence of hydroxyapatite in the rhizosphere soil induced the lead that migrated throughout the rhizosphere soil profile to be insoluble. Furthermore, the lead did not accumulate as the exchangeable phase in the rhizosphere soil that shares a boundary with the root surface. Thus, plant growth of hairy vetch was not suppressed as compared with that in the soil without the hydroxyapatite and the lead concentrations of the plant shoots remained at background levels in the presence of hydroxyapatite. These results indicate that the presence of hydroxyapatite in the rhizosphere soil at 5% wt could immobilize most of the lead migrating from contaminated soil in the rhizosphere soil within 1 mm from the contaminated soil, resulting in the prevention of lead migration toward the root surface. Thus, plants could grow without the adverse effects of lead toxicity. However, even in the rhizosphere soil with the hydroxyapatite, a small amount of lead migrated and accumulated as the water-extractable phase into the rhizosphere soil that shares a boundary with the root surface. Thus, it is suggested that, in cases where plants with a low tolerance to lead toxicity must be selected, the dosage of material should be increased in order to strictly prevent lead migration in the rhizosphere soil.

## Figures and Tables

**Table 1:** Physicochemical properties of soils used in this study (on the basis of air-dried weight).

Soil	pH	TC ^a^	Water-extractable		Total		Amorphous Fe (g kg^−1^)
		(g kg^−1^)	OC ^b^	Pb	Pb	P	
			(mg kg^−1^)	(mg kg^−1^)	(g kg^−1^)	(g kg^−1^)	
Contaminated soil	7.2	51	354	11.2	25.7	0.40	2.6
Non-contaminated soil	5.3	16	99	<0.02	0.02	0.12	3.0

^a^ Total carbon; ^b^ Organic carbon.

**Table 2:** Shoot biomass, lead concentration, and amount of lead uptake by plants grown in the rhizobox system (*n* = 3).

Plant	Material	Shoot Weight	Lead Concentration	Lead Uptake
		(a)	(b)	(c = a × b)
		(mg box^−1^ DW)	(μg g^−1^ DW)	(μg box^−1^ DW)
Barley	+HAP	410 ± 90	7 ± 2	2.7 ± 0.2
	−HAP	240 ± 40	90 ± 70	18 ± 13
*p* value		0.1513	0.2964	0.2867
Hairy vetch	+HAP	700 ± 100	9 ± 1	6.1 ± 1.2
	−HAP	244 ± 24	24 ± 7	5.5 ± 1.3
*p* value		0.0014 **	0.1005	0.7530

DW: dried-weight; +HAP: with hydroxyapatite; −HAP: without hydroxyapatite; ** *p* value < 0.01.

**Table 3:** Effect of material on soil pH in rhizosphere (*n* = 3).

Soil	Barley		Hairy Vetch	
	+HAP	−HAP	+HAP	−HAP
RC1	6.2 ± 0ab	5.2 ± 0a	6.2 ± 0a	5.2 ± 0a
RC2	6.3 ± 0a	5.1 ± 0a	6.2 ± 0a	5.0 ± 0b
RC3	6.1 ± 0b	5.0 ± 0a	6.1 ± 0b	4.9 ± 0c

Values for each plant with the same letter within each column are not significantly different (*p* < 0.05).
